# RhoGDIα suppresses self-renewal and tumorigenesis of glioma stem cells

**DOI:** 10.18632/oncotarget.11423

**Published:** 2016-08-19

**Authors:** Fan Wu, Peishan Hu, Dengke Li, Yan Hu, Yingjiao Qi, Bin Yin, Tao Jiang, Jiangang Yuan, Wei Han, Xiaozhong Peng

**Affiliations:** ^1^ State Key Laboratory of Medical Molecular Biology, Department of Molecular Biology and Biochemistry, Institute of Basic Medical Sciences Chinese Academy of Medical Sciences, School of Basic Medicine Peking Union Medical College, Beijing 100005, China; ^2^ Department of Neurosurgery, Beijing Tiantan Hospital, Beijing 100050, China

**Keywords:** GSCs, RhoGDIα, self-renewal, ROCK1, Oct4

## Abstract

Glioma stem cells (GSCs) are a subset of tumor cells that drive glioma initiation and progression. The molecular mechanisms underlying the maintenance of GSCs are still poorly understood. Here we investigated the role of Rho GDP dissociation inhibitor α (RhoGDIα) in GSCs. RhoGDIα was down-regulated in glioma stem cells. Over-expression of RhoGDIα suppressed the self-renewal and tumorigenesis of GSCs. Further data showed that RhoGDIα inhibited the transcription activity of stem cell marker Oct4. Moreover, inactivation of ROCK1, a downstream effector of RhoGDIα, also decreased the self-renewal and Oct4 transcription activity, and rescued the effects caused by RhoGDIα knockdown. Our results indicate that RhoGDIα is involved in the maintenance of GSCs.

## INTRODUCTION

Glioma is the most common and lethal primary brain tumor with few advances in treatment. Despite of current standard therapy, the life expectancy of patients with glioblastma (GBM) is about 14 months [1]. Recently, increasing evidences revealed that glioma contains a minor of cells referred to as glioma stem cells(GSCs), which are characterized by their self-renewal potential and strong tumorigenic capacity [2–4]. Moreover, GSCs have been shown to be more resistant to conventional chemotherapy and radiotherapy, and responsible for glioma recurrence [5–7]. Therefore, a novel therapeutic strategy that directly targets and eradicates GSCs was proposed. However, the understanding of the biology of GSCs remains partial, and the regulation mechanism of stemness maintenance and tumorigenesis needs further study.

Rho GDP dissociation inhibitors (RhoGDIs) are important regulators of the Rho family of small GTPases which control a wide range of biological processes, including cell adhesion, migration, apoptosis and proliferation [8–10]. RhoGDIα, also known as RhoGDI1, is the best characterized member of the family and ubiquitously expressed in mammalian organs [11]. Accumulating studies show that RhoGDIα regulates several processes during tumorigenesis and cancer progression, and its expression varies depending on the tumor types. The upregulation of RhoGDIα increased cell proliferation and migration in hepatocellular carcinoma [12], whereas the loss of RhoGDIα expression promotes the development and progression in prostate cancer [13]. In brain cancers, RhoGDIα expression is reduced and related with the decreased expression of RhoA and RhoB [14]. The new studies show that RhoGDIα interacts with αvβ8 integrin and Plexin-B3 to modulate glioma invasion by mediating activation of Rho proteins [15, 16]. Our recent study revealed that the interplay between PCBP2 and miRNA modulates RhoGDIα expression and function in glioma migration and invasion [17]. However, the function of RhoGDIα in cancer stem cells, particularly in GSCs, remains unclear.

The aim of this study was to determine the role of RhoGDIα in GSCs, and the possible mechanism involved was also investigated. The findings of this study may provide a basis for improving therapy against human glioma.

## RESULTS

### Isolation and identification of GSCs

First, we isolated two GSCs (named as GSC2 and GSC5) from human glioma samples (Figure S1A) (pathological data were shown in Table S2), which showed characteristics consistent with cancer stem cells. Both of them had the self-renewal capability proved by the limiting dilution assay. A single cell from the primary tumor spheres could form secondary tumor spheres (Figure S1B), and expressed stem cell makers: CD133, SOX2 or Nestin (Figure S1C). Moreover, Single-cell suspensions of spheres were subjected to differentiation assay and stained with markers for astrocyte (GFAP) and neuron (Tuj1) (Figure S1D), suggesting the potential for multilineage differentiation of GSCs. For *in vivo* tumor formation assay, hematoxylin & eosin (HE) staining showed GSC2 and GSC5 were both able to form brain tumor in nude mice (Figure S1E). In addition, we enriched two glioma stem-like cells from glioma cell lines as described previously [18]. U87MG stem-like cell (U87MG-SLC) and U251 stem-like cell (U251-SLC) were isolated from U87MG and U251 glioma cell, and cultured in Neurobasal medium (Figure S2A).

### The expression analysis of RhoGDIα in GSCs

**Figure 1:**
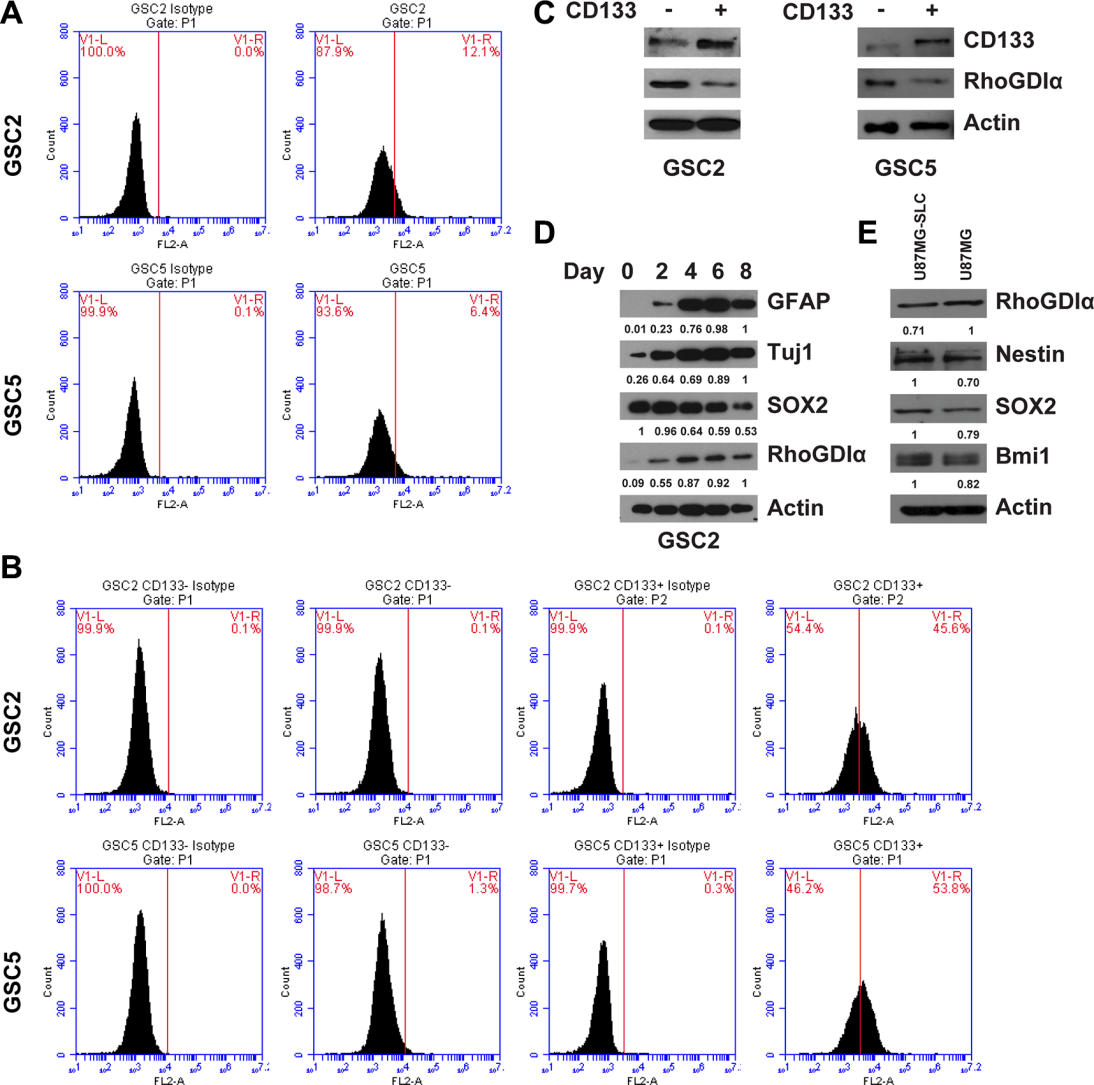
The RhoGDIα expression in GSCs (**A**) FASC analysis of CD133 percentage before magnetic sorting. (**B**) FASC analysis of CD133 percentage after sorting of CD133^−^ and CD133^+^ cells. CD133^+^ cells were isolated from GSC2 and GSC5 by magnetic beads, the percentage of CD133^+^ cells was determined by FASC analysis relative to cells labeled with IgG isotype control antibody. (**C**) Immunoblot analysis of RhoGDIα protein level in matched CD133^−^ and CD133^+^ cell isolated from GSC2 and GSC5. (**D**) Immunoblot analysis of RhoGDIα, stem cell marker (SOX2) and differentiation markers (GFAP and Tuj1) during GSC2 differentiation. The numbers represent gray value relative to Actin. (**E**) Immunoblot analysis of RhoGDIα and stem cell markers (Nestin, SOX2 and Bmi1) in U87MG and U87MG-SLC cell. The numbers represent gray value relative to Actin.

To confirm whether RhoGDIα was associated with GSCs maintenance, we first examined the expression of RhoGDIα in matched CD133^−^ and CD133^+^ cells sorted from GSC2 and GSC5. CD133^+^ and CD133^−^ cells were separated using a CD133 microbead kit. Before magnetic sorting, we analyzed the CD133 expression in GSC2 and GSC5 though FASC, and the percentage of CD133^+^ cells in GSC2 and GSC5 were 12.1% and 6.4% respectively (Figure 1A). After sorting, the percentage of CD133^+^ cells reached 45.6% and 53.8% in GSC2 and GSC5 CD133^+^ cells (Figure 1B). The western blot showed RhoGDIα protein level was commonly lower in CD133^+^ cells than matched CD133^−^ cells (Figure 1C). Next, we assessed the change in RhoGDIα expression as GSCs differentiate. Intriguingly, RhoGDIα, astrocyte marker GFAP and neuronal marker Tuj1 gradually increased, whereas the expression of GSC marker SOX2 decreased during GSC2 differentiation (Figure 1D). Furthermore, we compared the RhoGDIα expression between U87MG cell and U87MG-SLC. Compared with U87MG cell, U87MG-SLC showed higher expression level of stem cell markers Nestin, SOX2 and Bmi1, but lower RhoGDIα expression (Figure 1E). Thus, these findings suggest that decreased expression of RhoGDIα may be a distinctive feature of GSCs.

### RhoGDIα suppressed stemness and self-renewal ability of GSCs

**Figure 2:**
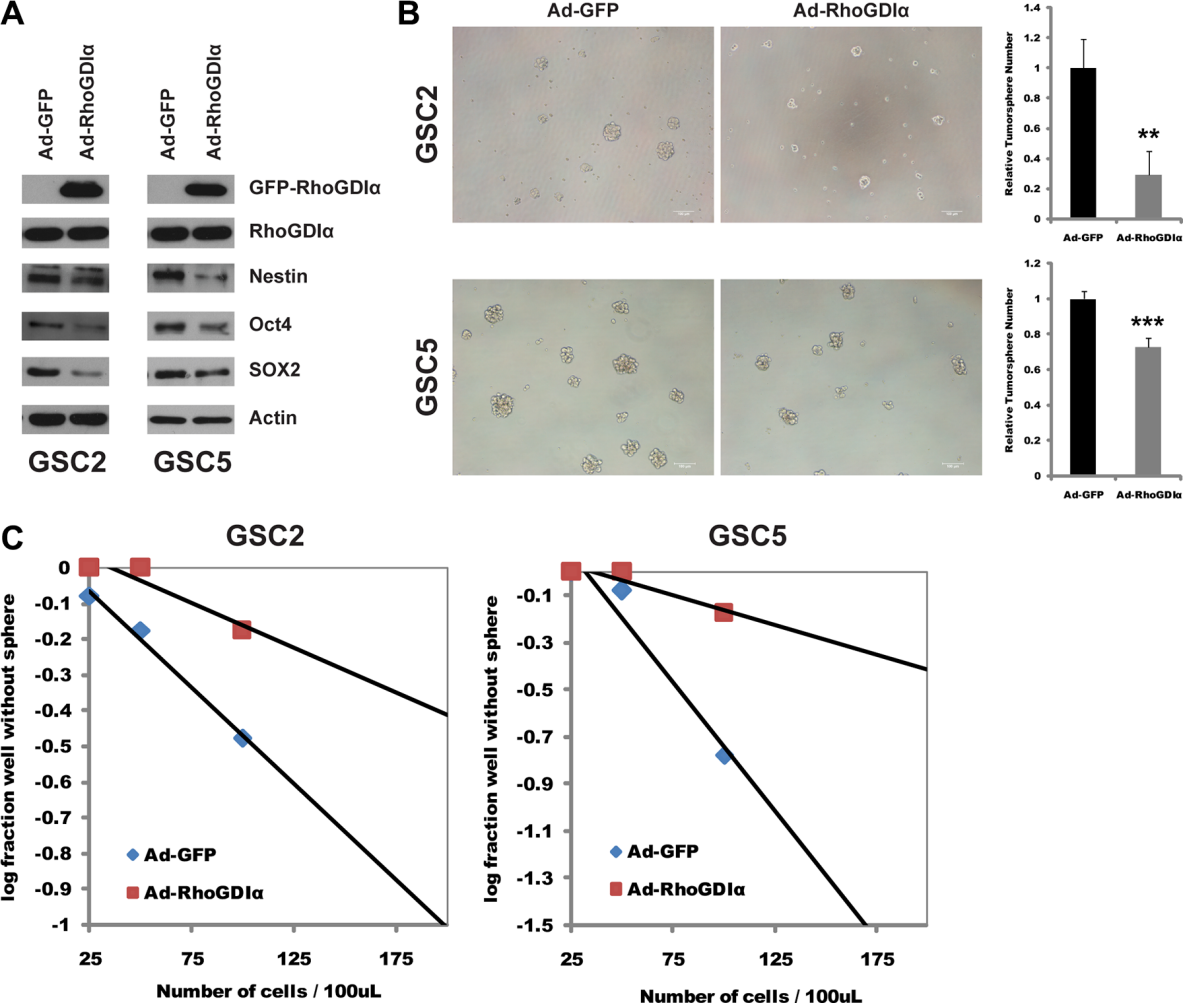
RhoGDIα suppressed stemness and self-renewal ability of GSCs (**A**) Immunoblot analysis of stem cell markers (Nestin, Oct4 and SOX2) in GSCs infected with Ad-RhoGDIα and Ad-GFP adenovirus (control). (**B**) Sphere formation assay in GSC2 and GSC5 cells infected with Ad-RhoGDIα and Ad-GFP adenovirus (control). Data are means ± SD, **p < 0.01, ***p < 0.001. Scale bars represent 100 μm. (**C**) Limiting dilution neurosphere assay in GSC2 and GSC5 cells infected with Ad-RhoGDIα and Ad-GFP adenovirus (control).

To define the functional significance of the decreased expression of RhoGDIα in GSCs, we performed over-expression assay and examined the effects on the stemness and self-renewal of GSCs. RhoGDIα over-expression caused a significant decrease in protein level of stem cell markers Nestin, Oct4 and SOX2 in all of 4 cells (Figure 2A, Figure S2B). Similarly, the spheres number and formation efficiency were both reduced markedly (Figure 2B and 2C, Figure S2C and S2D). These results demonstrate the inhibitory effects of RhoGDIα on GSCs stemness and self-renewal capacity.

### RhoGDIα reduced GSCs tumorigenic potential

**Figure 3:**
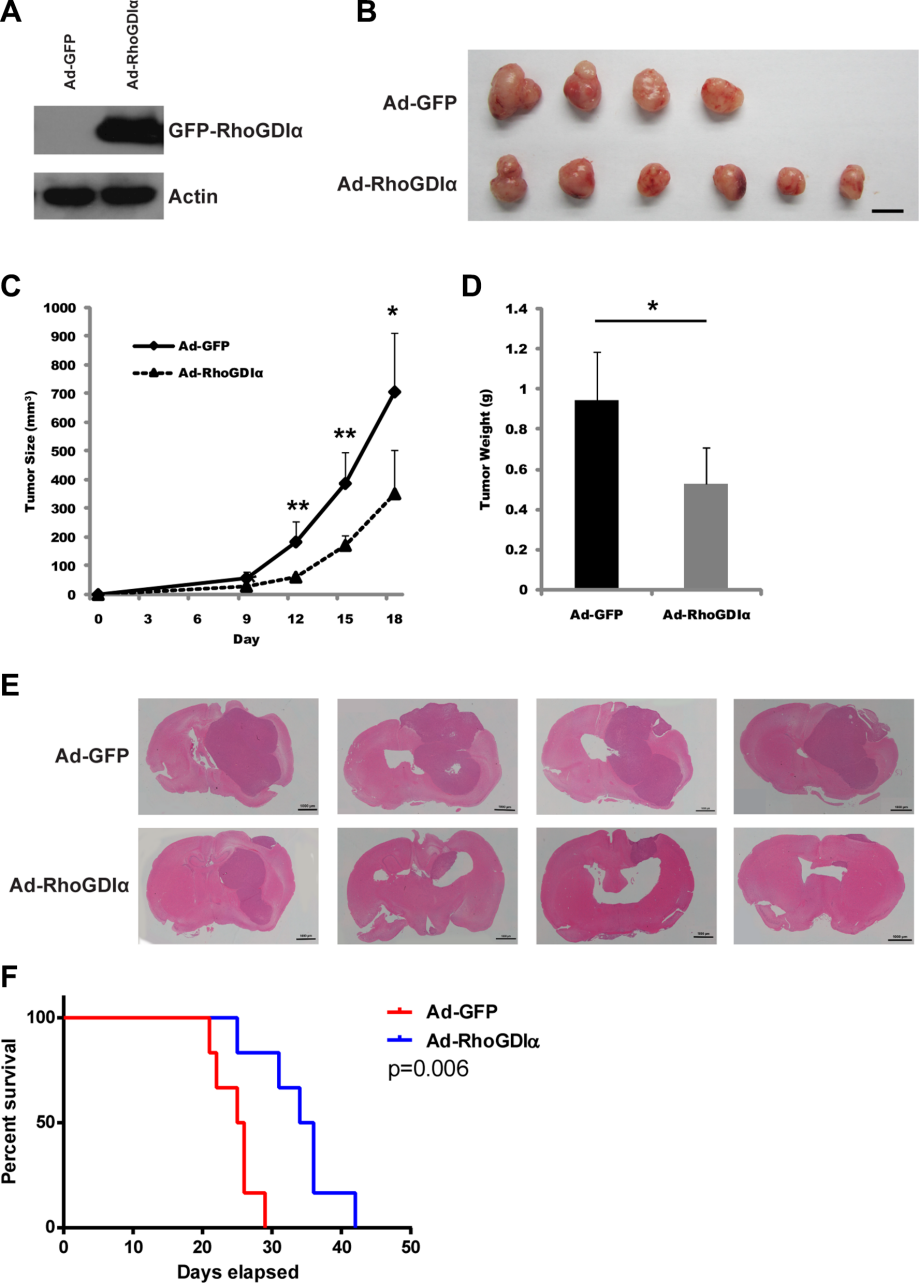
RhoGDIα reduced GSCs tumorigenic potential (**A**) Immunoblot analysis of RhoGDIα protein levels in U87MG-SLC cell after infected with Ad-RhoGDIα and Ad-GFP adenovirus. (**B**) Tumors isolated from nude mice of Ad-RhoGDIα (*n* = 6) and Ad-GFP group (*n* = 4). Scale bars represent 1 cm. (**C**), (**D**) Tumor size and weight in nude mice after injected with U87MG-SLC. Data are means ± SD, *p < 0.05, **p < 0.01, ***p < 0.001. (**E**) 5 × 10^5^ U87MG-SLC cells infected with Ad-RhoGDIα or Ad-GFP adenovirus were each injected intracranially into 4 nude mice, and HE staining was using to detect the tumors after 20 days. (**F**) Overall survival was determined by Kaplan-Meier survival curves. *n* = 6, Log-rank test.

Since RhoGDIα is critical to GSCs self-renewal capacity, we examined whether ectopic expression of RhoGDIα impacts the tumorigenicity of GSCs. U87MG-SLC cell was chosen for its high infection efficiency of adenoviruses. After infected with Ad-GFP and Ad-RhoGDIα adenoviruses for 3 days, the over expression of RhoGDIα was detected (Figure 3A). Subsequently, the infected U87MG-SLC cells were injected in the right axilla of nude mice subcutaneously. As a result, the mice in Ad-RhoGDIα group developed much smaller tumors compared with the mice in Ad-GFP group (Figure 3B). The tumor size and weight in Ad-RhoGDIα group were also decreased (Figure 3C and 3D). We then proceeded to examine the effect of RhoGDIα over-expression in an orthotopic tumor growth model. 5 × 10^5^ U87MG-SLC cells infected with Ad-GFP or Ad-RhoGDIα adenoviruses were injected intracranially, and the tumor size was detected by HE staining. The RhoGDIα over-expression group showed markedly suppressed tumor formation in mice (Figure 3E). Furthermore, the overall survival time of xenograft mice was prolonged in Ad-RhoGDIα group (*p* < 0.01, Figure 3F). Collectively, these data indicate that over-expression of RhoGDIα reduced the tumor formation of GSCs.

### RhoGDIα negatively regulated the transcription of stem cell marker Oct4

**Figure 4:**
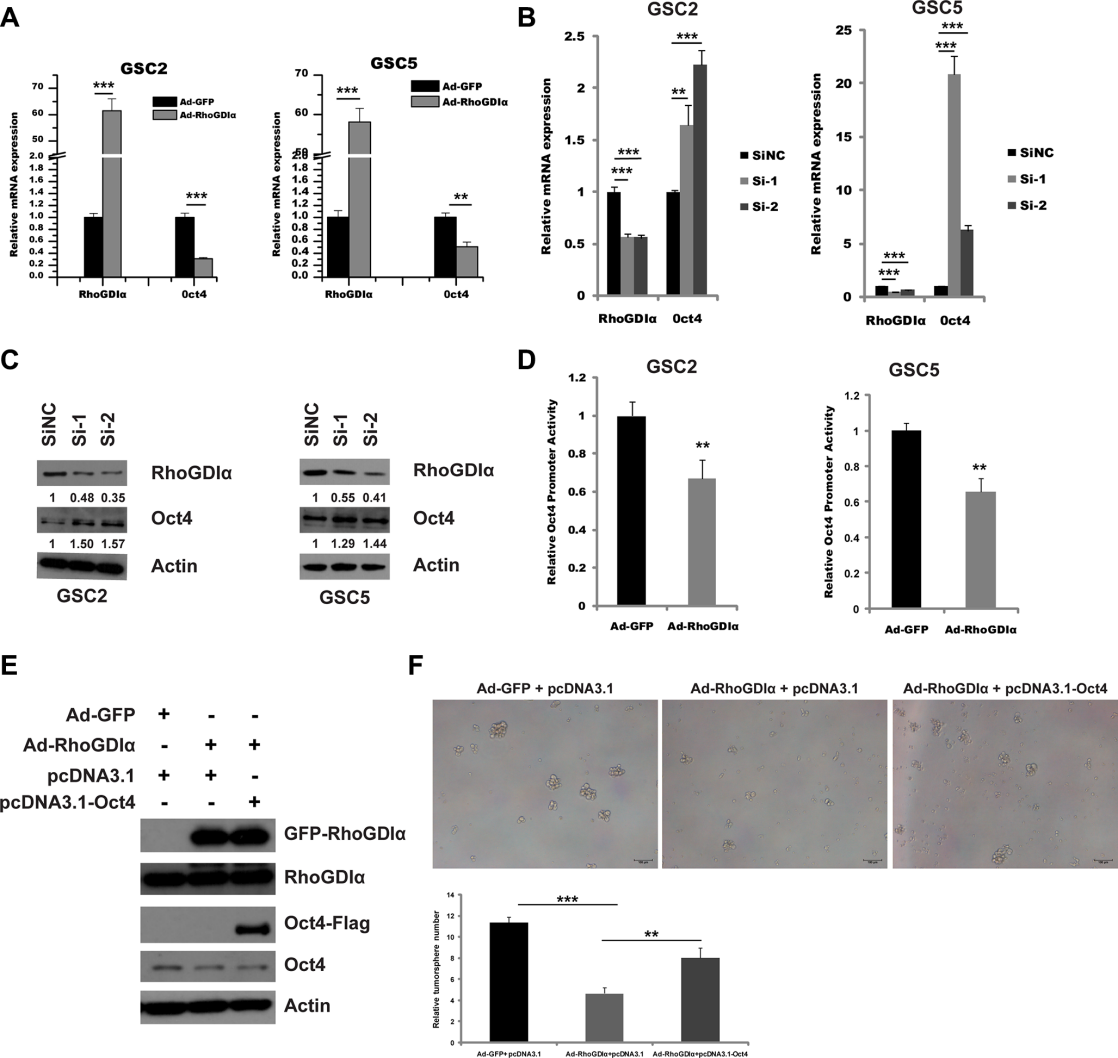
RhoGDIα suppressed Oct4 transcription (**A**) qRT-PCR analysis of stemness-associated gene Oct4 in GSC2 and GSC5 infected with Ad-RhoGDIα and Ad-GFP adenovirus (control). (**B**) qRT-PCR analysis of stemness-associated gene Oct4 in GSC2 and GSC5 transfected with RhoGDIα siRNAs or siNC (control). (**C**) Immunoblot analysis of Oct4 protein levels in GSC2 and GSC5 transfected with RhoGDIα siRNAs or siNC (control). The numbers represent gray value relative to Actin. (**D**) Analysis of Oct4 promoter activity by luciferase reporter assay in GSCs in response to RhoGDIα ectopic expression. (**E**) Western blot analysis of RhoGDIα and Oct4 protein levels in GSC5 infected with Ad-RhoGDIα alone, or Ad-RhoGDIα plus pcDNA3.1-Oct4 transfection. (**F**) Tumorsphere formation assay of GSC5 after infection and transfection. Data are means ± SD, **p < 0.01, ***p < 0.001.

RhoGDIα is known to exert biological role by interacting with a variety of protein molecular in cancers [19–21]. To further understand the mechanism how RhoGDIα negatively regulates the GSCs maintenance, we investigated the interaction between RhoGDIα and stem cell markers. Unfortunately, no apparent immunoprecipitation was identified (data not shown). Next, we measured the mRNA level of several genes related to self-renewal and differentiation, including SOX2, Oct4, Bim1, Nestin and CD133. Intriguingly, Oct4 expression was significantly decreased by RhoGDIα over-expression (Figure 4A). Meanwhile, RhoGDIα knockdown resulted in a remarkable increase in Oct4 expression level, whereas the expression of other genes showed minor alteration (Figure 4B). Western blot also showed an increase in protein level of Oct4 after RhoGDIα knockdown (Figure 4C). Therefore, we further figured out whether RhoGDIα inhibits the expression of Oct4 at transcriptional level. We cloned human Oct4 promoter into the luciferase report system (Figure S3) and tested promoter activity in response after RhoGDIα over-expression. Forced expression of RhoGDIα significantly decreased Oct4 promoter activity in GSCs (Figure 4D). To further test that RhoGDIα regulates the self-renewal capacity of GSCs by suppressing the expression of Oct4, we performed a co-overexpression of RhoGDIα and Oct4 as a rescue assay. Western blot analysis revealed that RhoGDIα and Oct4 were co-overexpressed into GSC5 cell (Figure 4E). The tumorsphere formation assay demonstrated that the number of tumorsphere increased distinctly by co-overexpression comparing with RhoGDIα overexpression alone (Figure 4F). Taken together, these results support the hypothesis that RhoGDIα modulates the self-renewal of GSCs by suppressing the transcription activity of stemness related gene Oct4.

### ROCK1 inhibitor (Y27632) decreased the transcription of Oct4 and self-renewal ability and rescued the effects of RhoGDIα knockdown

**Figure 5:**
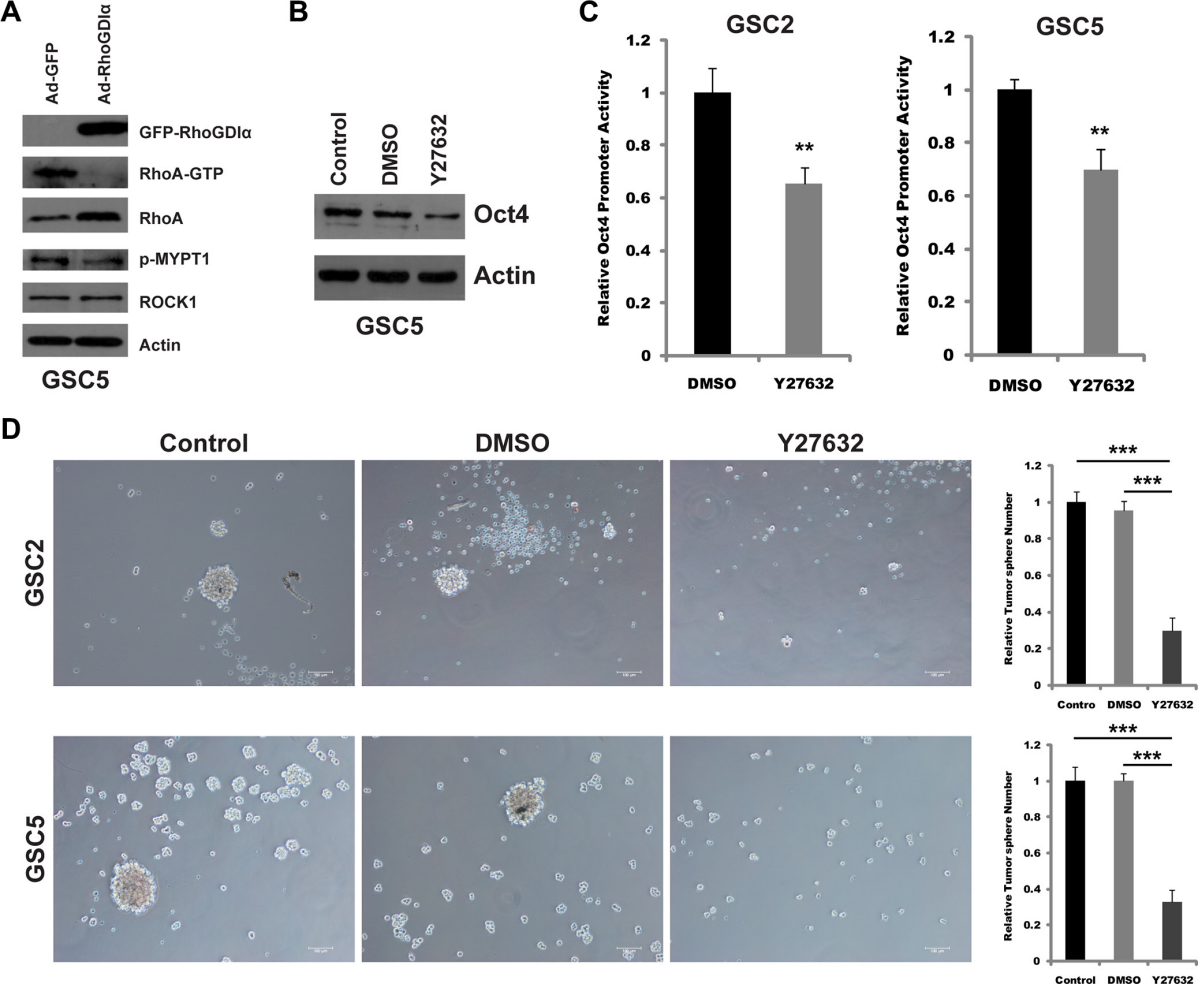
Y27632 inhibited the transcription of Oct4 and self-renewal ability (**A**) Immunoblot analysis of the expression and activity of RhoA and ROCK1 in GSC5 infected with Ad-RhoGDIα and Ad-GFP adenovirus. (**B**) Immunoblot analysis of Oct4 protein levels in GSC5 in response to ROCK1 inhibitor Y27632 (10 μM). (**C**) Analysis of Oct4 promoter activity by luciferase reporter assay in GSCs treated with ROCK1 inhibitor Y27632 (10 μM). (**D**) Sphere formation assay in GSC2 and GSC5 treated with ROCK1 inhibitor Y27632 (10 μM). Scale bars represent 100 μm. Data are means ± SD, *p < 0.05, **p < 0.01, ***p < 0.001.

RhoA/ROCK1 is known as a downstream pathway of RhoGDIα in many cancers [22–24]. Therefore, we presumed that the RhoGDIα might affect Oct4 transcription and self-renewal by RhoA/ROCK1 pathway in GSCs. As expected, Ectopic expression of RhoGDIα inhibited RhoA activity. Consistently, the phosphorylated level of myosin phosphatase (MYPT1) which is associated with the activity of ROCK1 also decreased (Figure 5A). Inactivation of ROCK1 by special inhibitor (Y27632) reduced the protein level and promoter activity of Oct4 in GSC5 (Figure 5B and 5C). Likewise, blocking ROCK1 decreased the tumorsphere formation in GSCs (Figure 5D).

**Figure 6:**
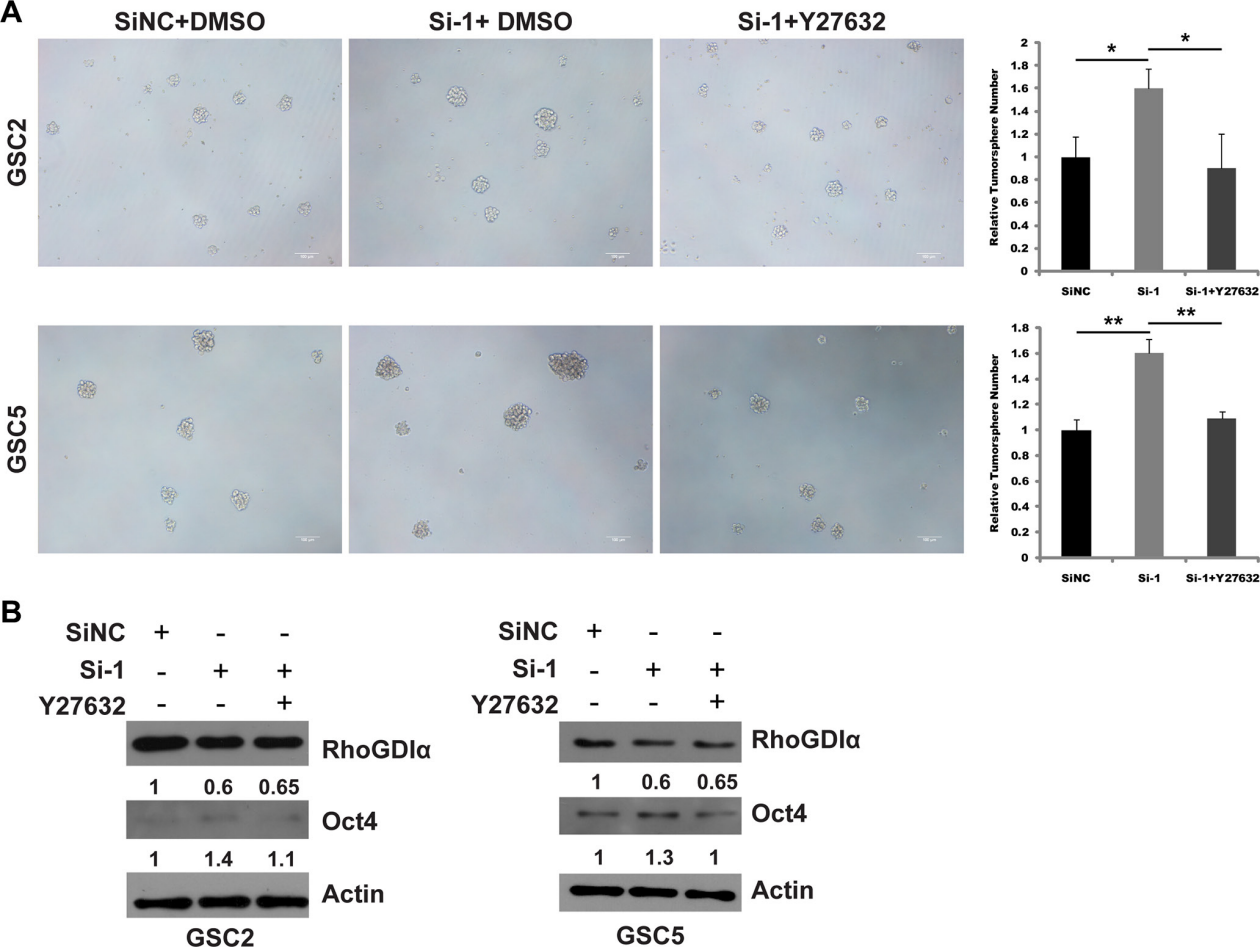
ROCK1 inhibition rescued the effects of RhoGDIα knockdown in GSCs (**A**) Sphere formation assay in GSC2 and GSC5 transfected with siNC or si-1 and treated with DMSO or Y27632. (**B**) Immunoblot analysis of Oct4 expression in GSC2 and GSC5 transduced with siNC or si-1 and treated with DMSO or Y27632. The numbers represent gray value relative to Actin. Scale bars represent 100 μm. Data are means ± SD, *p < 0.05, **p < 0.01.

To confirm RhoA/ROCK1 as a critical molecular mediating the effects of RhoGDIα on self-renewal, we examined whether ROCK1 inhibition could rescue the effects caused by RhoGDIα knockdown. GSCs were transduced with siNC or si-1 and treated with DMSO or Y27632. Consequently, Y27632 attenuated the increased tumorsphere formation of GSCs transfected with siRNA (Figure 6A). Consistently, Immunoblot analysis also showed Y27632 was able to reduce the protein level of Oct4 caused by RhoGDIα knockdown (Figure 6B). These findings revealed RhoA/ROCK1, as a downstream of RhoGDIα, is involved in regulating GSCs' self-renewal.

## DISCUSSION

In this study, we revealed the effects of RhoGDIα on GSCs, self-renewal and tumorigenesis. Interestingly, RhoGDIα was preferentially expressed in CD133^−^ cells than CD133^+^ cells. During GSC differentiation, the expression of RhoGDIα gradually increased, which implied an inhibitory role in GSC maintenance. Over expression of RhoGDIα suppressed the stemness and self-renewal ability, and reduced *in vivo* tumor formation of GSCs. Oct4 is best known as a regulator of self-renewal and differentiation in GSCs [25]. We found RhoGDIα regulated the GSCs self-renewal and inhibited Oct4 transcription through RhoA/ROCK1 pathway.

RhoGDIα plays a critical role in modulating cell proliferation, migration, invasion in cancers [12–16]. However, the functional role of RhoGDIα in stem cells, such as in embryonic stem cells and neural stem cells (NSCs), is poorly understood, let alone in GSCs. The family member RhoGDIγ maintains neural stem cell phenotypes and inhibits its migration. Down-regulation of RhoGDIγ promotes the differentiation of NSC [26, 27]. We first determined the functional significance of RhoGDIα in maintenance of GSCs, and found over-expression of RhoGDIα in GSCs markedly inhibited the stemness and self-renewal ability. To test whether RhoGDIa is an inhibitory factor for self-renewal and stemness maintenance definitively, we conducted the knockdown study in U87MG-SLC (low expression of CD133), and the results showed RhoGDIα knockdown by siRNAs increased the protein level of stem cell markers Oct4, SOX2 and Nestin (Figure S4A and S4B). Furthermore, we performed the knockdown assay in GSC5 CD133- cells. The tumorsphere numbers and the expression level of stem cell markers increased similarly (Figure S4C and S4D). Since overexpression of RhoGDIα inhibits glioma migration and invasion, we also examined the effect of RhoGDIα on GSCs. Using *in vitro* transwell assay, the migration ability of U87MG-SLC and GSC5 cells was significantly inhibited in the Ad-RhoGDIα group (Figure S5A). A similar effect on invasion ability was also observed; RhoGDIα greatly reduced the number of invasive cells (Figure S5B). Furthermore, glioma stem cells have been implicated in the resistance to the traditional chemo- and radiotherapy [5–7]. To examine whether RhoGDIα is involved in the resistance of GSCs to anticancer treatment, we exposed GSCs to the methylating brain tumor drug temozolomide (TMZ). RhoGDIα over-expression cells showed distinctly inhibitory growth compared to the controls at different concentrations (Figure S5C). The Rho family of small GTPases, regulated by RhoGDIs, is implicated in several biological processes in many cancers, including cell migration, invasion, apoptosis and proliferation. Previous studies have suggested that Rho GTPases also play an important role in regulating cancer stem cell. In breast cancer stem cell (BCSC), RhoC is an important regulator of BCSC metastasis and its expression is highly correlative with BCSC marker ALDH1 [28]. Blockade of Rac1 suppresses the CSC proliferation and metastasis in human non-small cell lung adenocarcinoma [29]. In glioma, Rac1 is involved in stemness maintenance, surviving and apoptosis of GSCs [30, 31]. Our study also extended the understanding of Rho GTPases in GSCs. RhoA/ROCK pathway repressed by ectopic expression of RhoGDIα might modulate the stemness maintenance of GSCs.

Rho-associated protein kinase1 (ROCK1), a key downstream effector of the small GTPase RhoA, is a member of Rho-associated serine/threonine kinase family [32]. Recent studies showed that ROCK1 functions as an oncogene and possesses a wide range of functions in cancers, such as cell motility, apoptosis, survival, proliferation and metastasis [33–35]. In glioma, Oellers et al. found that ROCK1 is highly expressed in human high grade glioma, and required for migration [36]. A new study has reported knockdown of ROCK1 suppresses proliferation and invasion of glioma cells [37]. However, reports of ROCK1 in cancer stem cells are very rare. Hirokazu et al. found that ROCK1 inhibitors improved the sphere-formation efficiency from primary colon cancer cells [38]. In mouse mammary cancer stem cells, ROCK1 inhibition promoted the self-renewal ability [39]. Conversely, our study unveiled that ROCK1 inhibition reduced the self-renewal in glioma stem cells, which suggests the function of ROCK1 may differ in different cancer stem cells.

In summary, we have identified RhoGDIα/RhoA/ROCK1 pathway as a regulator of GSCs maintenance, and RhoGDIα is a potential molecular target of GSCs for future therapy of glioma.

## MATERIALS AND METHODS

### Tumor samples

Patient specimens were collected from the department of neurosurgery of Tiantan Hospital following written informed consent and the institutional review board of Beijing Tiantan Hospital approval. The pathological diagnosis was established according to the WHO classification.

### Isolation, culture and identification of GSCs

Briefly, tumor tissue was washed and dissociated enzymatically as previously described [39]. The single cell suspension was cultured in Neurobasal medium (GIBCO) supplemented with 20 ng/ml of basic fibroblast growth factor (bFGF, Peprotech), 20 ng/ml of epidermal growth factor (EGF, Peprotech), 2% B27 (GIBCO), 10 μg/ml heparin (Sigma), and incubated at 37°C in an atmosphere with 5% CO_2_. To assess the multipotency of GSCs, cells were plated onto glass coverslips coated with poly-lysine (Sigma) and laminin (Invitrogen) in the cultural medium with 10% fetal bovine serum (FBS) for 1 to 2 weeks. Inmmunostaining was performed with anti-GFAP (Abcam) and anti-Tuj1 (Abcam). Anti-Nestin (Millipore), anti-SOX2 (Abcam) and anti-CD133 (Miltenyi) were used to stain the undifferentiated spheres. The cells were counterstained with DAPI to indentify all nuclei. To determine the tumorigenicity, 5,000 cells were injected into the right striatum of Balb/c athymic nude mice.

### Enrichment for glioma stem-like cells

Human glioblastoma cell line U87MG and U251 purchased from the American Type Culture Collection (ATCC) were cultured in Dulbecco's modified Eagle medium (DMEM) containing 10% FBS, 100 U/ml penicillin and 100 mg/ml streptomycin at 37°C. To isolate glioma stem-like cells, U87MG and U251 cell lines were suspended in neurobasal medium for spheres formation. These spheres were collected and passaged for future use, and named as U87MG-SLC and U251-SLC respectively.

### Cell sorting and flow cytometry

GSCs were dissociated into single cells followed by been suspended in phosphate-buffered saline (PBS) containing 0.5% bovine serum albumin (BSA) and 2mM EDTA. CD133^+^ and CD133^−^ cells were separated using a CD133 microbead kit (Miltenyi). The purity of the sorted cells was verified by flow cytometry with a CD133/2-PE antibody (Miltenyi). The sorted CD133^+^ cells were maintained in Neurobasal medium, CD133^−^ cells were cultured in DMEM with 10% FBS.

### Limiting dilution assay and sphere formation assay

Limiting dilution assay was performed as described previously [39]. In brief, the single-cell suspension were diluted and plated at 1–2 cells/well, cultures were fed 20 μl of medium every 3 days. For sphere formation assay, cells with indicated treatment were dissociated and seeded in 96 or 24-well plates at a density of 200 or 2,000 cells per well with 4 replicate wells for each group. After 7 to 10 days, the spheres with diameter > 100 μm were counted in each well under an inverted microscope (Nikon).

### RhoA activation assay

RhoA activation was determined using Rho activation assay kit (Millipore). Briefly, the total cell lysate was immunoprecipitated with Rhotekin RBD agarose bead at 4°C for 3 hours. After washing 3 times with lysis buffer, the GTP bound RhoA protein was eluted by boiling in sample buffer and evaluated by western blot.

### Reporter assay

The human Oct4 promoter region (−2601/−1) was amplified by PCR (see Table S1 for primer sequences) using human genomic DNA as the template, and cloned into pGL3-basic vector (Promega) containing a firefly luciferase reporter gene to get pGL3-Oct4 construct. 293ET or GSCs were co-transfected with 0.2 μg pGL3-Oct4 along with 50ng pRL-TK (Promega) using FuGENE 6 reagent (Promega). After 48 hours, the luciferase activity was measured with the dual-luciferse assay system according to the manufacturer's instructions (Promega).

### Quantitative RT-PCR

Total mRNA was purified from GSCs using Trizol reagent (Invitrogen). mRNA was reverse transcribed into cDNA with TransScript First-Strand cDNA Synthesis SuperMix (TransGen). Quantitative RT-PCR assay was performed with SYBR Premix EX Taq (TaKaRa). GAPDH was used as the internal reference and fold changes of gene expression levels were calculated by relative quantification (2^−ΔΔCt^) method (see Table S1 for primer sequences).

### Western blot analysis

Western blotting was performed according to the standard procedures. Antibodies used were as followed: anti-Nestin (1:2500, Millipore), anti-CD133 (1:200, Miltenyi), anti-SOX2 (1:500, Abcam), anti-Oct4 (1:500, Abcam), anti-Bmi1 (1:1000, Cell Signaling), anti-RhoGDIα (1:1000, Santa Cruz), anti-RhoA (1:1000, Cell Signaling), anti-p-MYPT1 (1:200, Cell Signaling), anti-ROCK1 (1:1000, Santa Cruz).

### Cell transfection and infection

RhoGDIα siRNAs (see Table S1 for sequences) were purchased from GenePharma and introduced into cells using INTERFERin reagent (Polyplus-transfection) according to the manufacturer's instrument. For infection, cells were maintained in the medium containing recombinant adenoviruses (Ad-RhoGDIα, Ad-GFP) packaged in GeneChem at a final concentration of 1 × 10^7^ pfu/ml.

### Xenograft model in nude mice

Animal experiments in this study were performed in accordance with protocols approved by the Institutional Animal Care and Use Committee at the Institute of Basic Medical Sciences Chinese Academy of Medical Sciences, China. Male BALB/c athymic nu mice (4-week-old, Vital River) used for *in vivo* assay were maintained at a temperature of 28°C and a relative humidity of 50% at no more than 5 mice per ventilated cage. U87MG-SLC cells infected with Ad-GFP or Ad-RhoGDIα (1 × 10^6^ cells in 100 μl physiological saline) were injected in the right frank of nude mice subcutaneously. The tumors were measured every 3 days thereafter. Tumor volume was estimated using the formula (V = L × W^2^ x π/6; V, volume; L, length; W, width).

### Statistics

Data were reported as means ± standard deviation (SD). Two-tailed student's *t* test was used to determine statistical significance. A *P* value of less than 0.05 was considered significant. The corresponding significance levels were indicated in figures.

## SUPPLEMENTARY MATERIALS FIGURES AND TABLES


