# Supplementary material for: RhoGDIα suppresses self-renewal and tumorigenesis of glioma stem cells
Source: Oncotarget. 2016 Aug 19;7(38):61619–29. doi: 10.18632/oncotarget.11423 (PMC5308677; doi:10.18632/oncotarget.11423)
Supplement: Supplementary file 1 [file oncotarget-07-61619-s001.pdf]

RhoGDIα suppresses self-renewal and tumorigenesis of glioma stem cells

Supplementary Materials

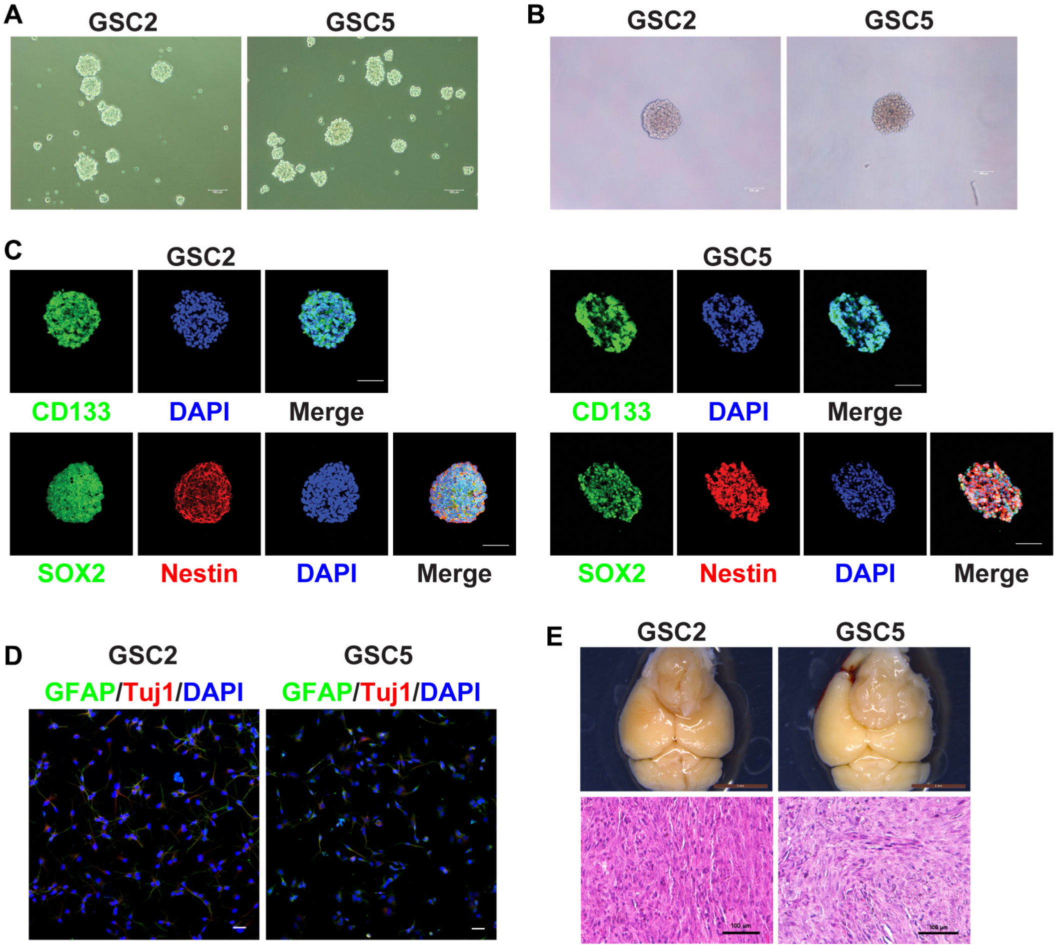

Supplementary Figure S1: Isolation and identification of GSCs. (**A**) GSC2 and GSC5 spheres were cultured and passaged in neurobasal medium. (**B**) GSC2 and GSC5 sphere generated from a single cell. Scale bars represent 100 µm. (**C**) Sphere was stained for CD133 (green), Nestin (red) and SOX2 (green) by immumofluorescence analysis. Nuclei were counterstained with DAPI (blue). Scale bars represent 100 µm. (**D**) Single-cell suspensions were differentiated and stained for GFAP (green) and Tuj1 (green). Nuclei were counterstained with DAPI (blue). Scale bars represent 100 µm. (**E**) 5,000 cells were injected into the right striatum of nude mice, the mice brains carrying tumors were shown, and the xenograft was stained with H&E. Scale bars represent 5 mm.

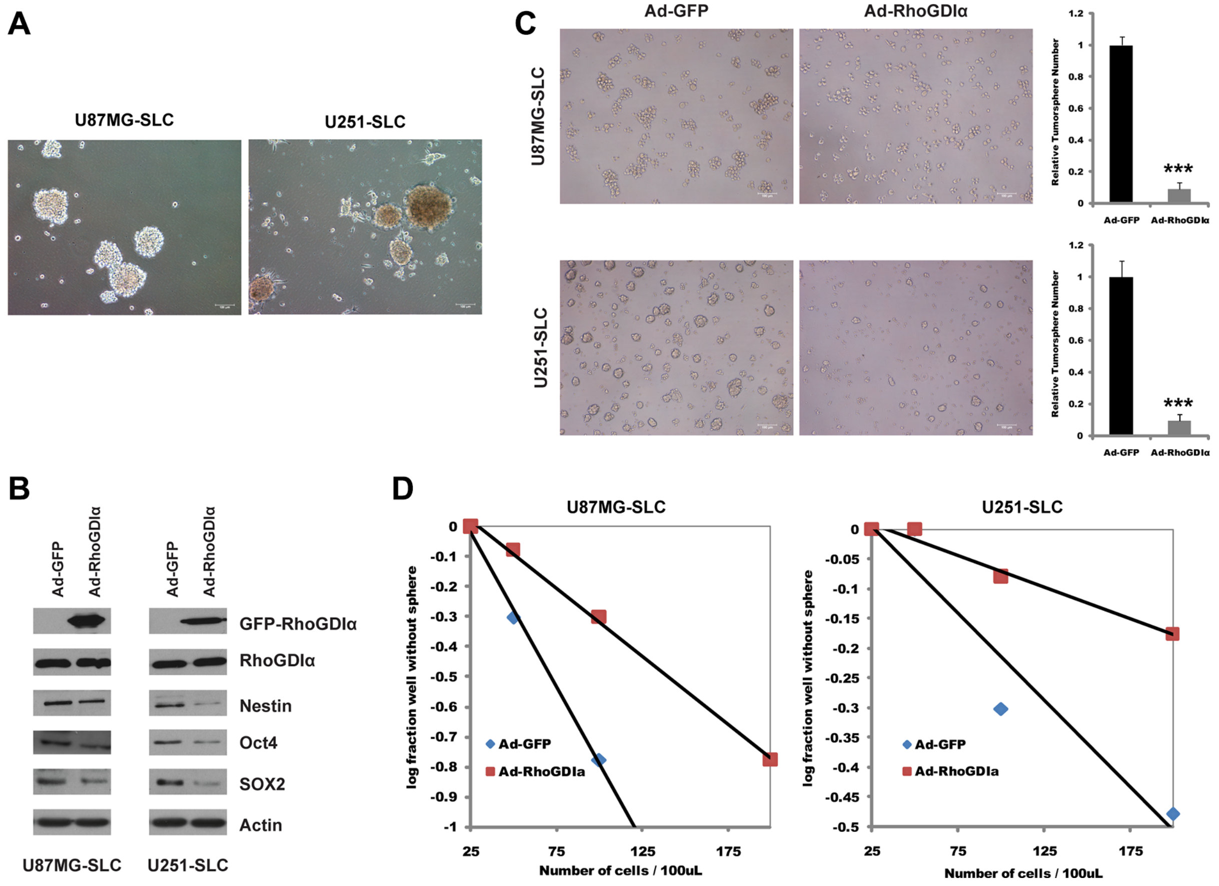

Supplementary Figure S2: RhoGDIα suppressed stemness and self-renewal ability of GSCs. (**A**) U87MG-SLC and U251-SLC were enriched from U87MG and U251 glioma cell, and cultured in neurobasal medium. (**B**) Immunoblot analysis of stem cell markers (Nestin, Oct4 and SOX2) in U87MG-SLC and U251-SLC infected with Ad-RhoGDIα and Ad-GFP adenovirus (control). (**C**) Sphere formation assay in U87MG-SLC and U251-SLC cell infected with Ad-RhoGDIα and Ad-GFP adenovirus (control). Data are means ± SD, ****p* < 0.001. Scale bars represent 100 μm. (**D**) Limiting dilution neurosphere assay in U87MG-SLC and U251-SLC cell infected with Ad-RhoGDIα and Ad-GFP adenovirus (control).

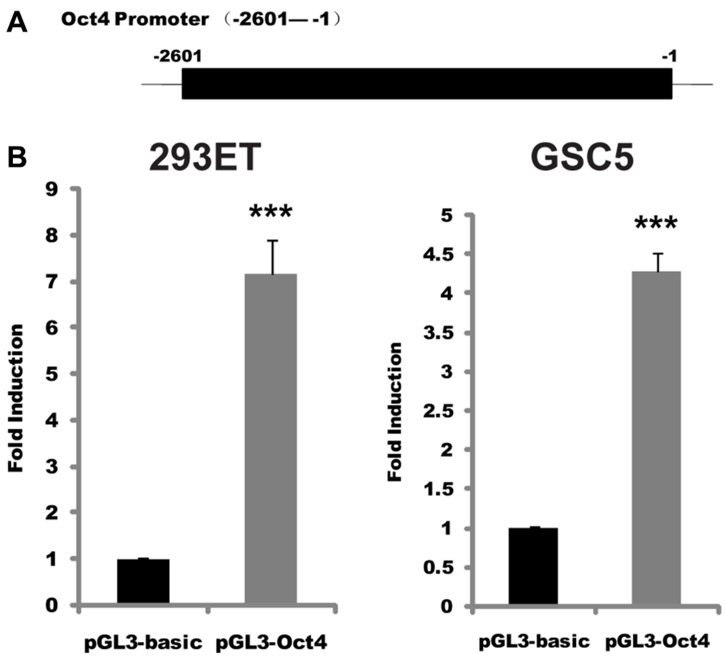

Supplementary Figure S3: (**A**, **B**) Human Oct4 promoter region (−2601 — −1) was cloned into pGL3-basic vector and the promoter activity was tested in 239ET and GSC5 cell. Data are means ± SD, ****p* < 0.001.

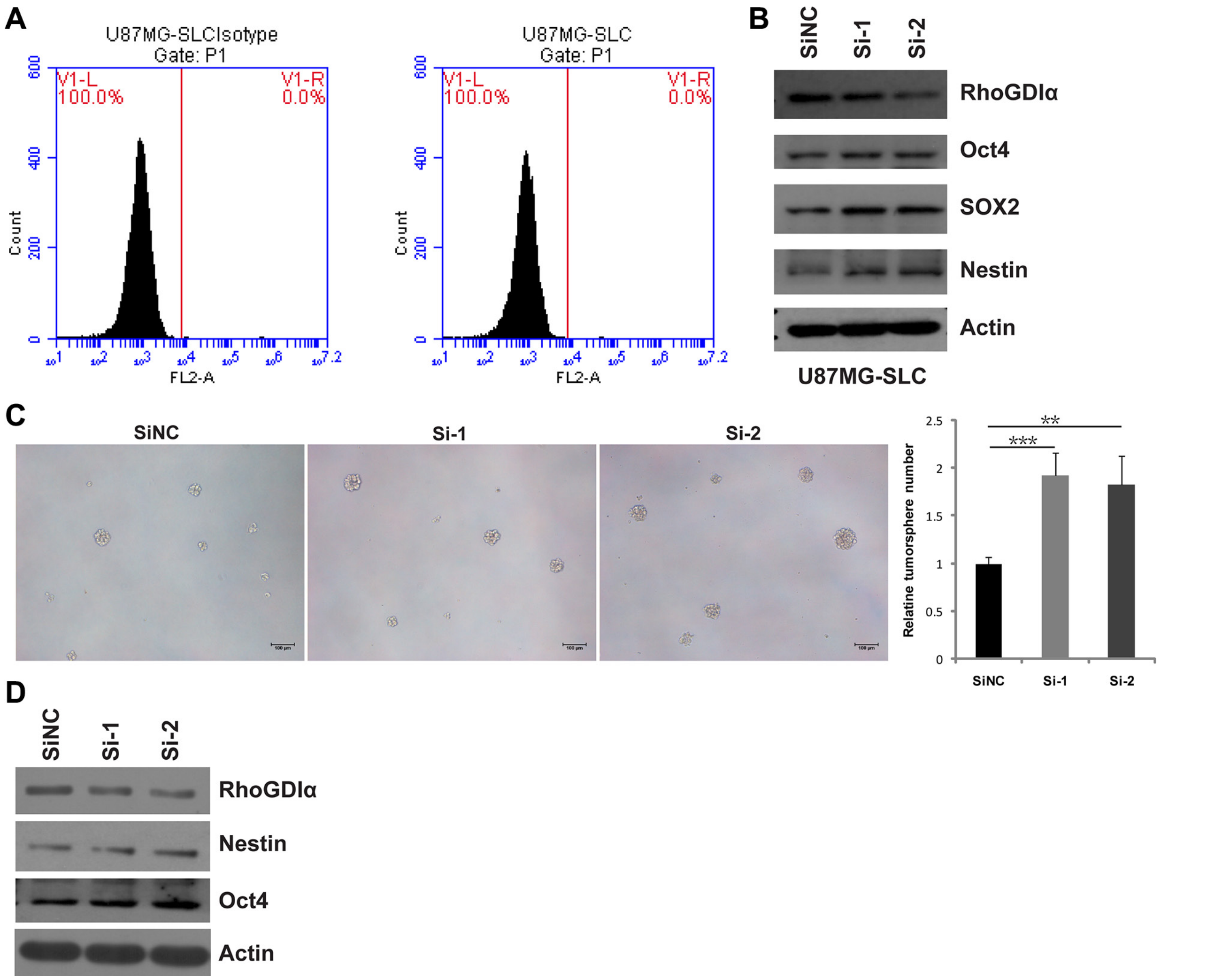

Supplementary Figure S4: RhoGDIα knockdown increased the self-renewal ability in U87MG-SLC and GSC5 CD133- cell. (**A**) FASC analysis of CD133 percentage in U87MG-SLC. (**B**) Immunoblot analysis of stem cell markers (Nestin, Oct4 and SOX2) in U87MG-SLC transfected with siRNAs and siNC (control). (**C**) Sphere formation assay in GSC5 CD133- cell transfected with siNC or si-1/2. Data are means ± SD, $***p < 0.001$. (**D**) Immunoblot analysis of stem cell markers (Nestin and Oct4) in GSC5 CD133- cell transfected with siRNAs and siNC (control).

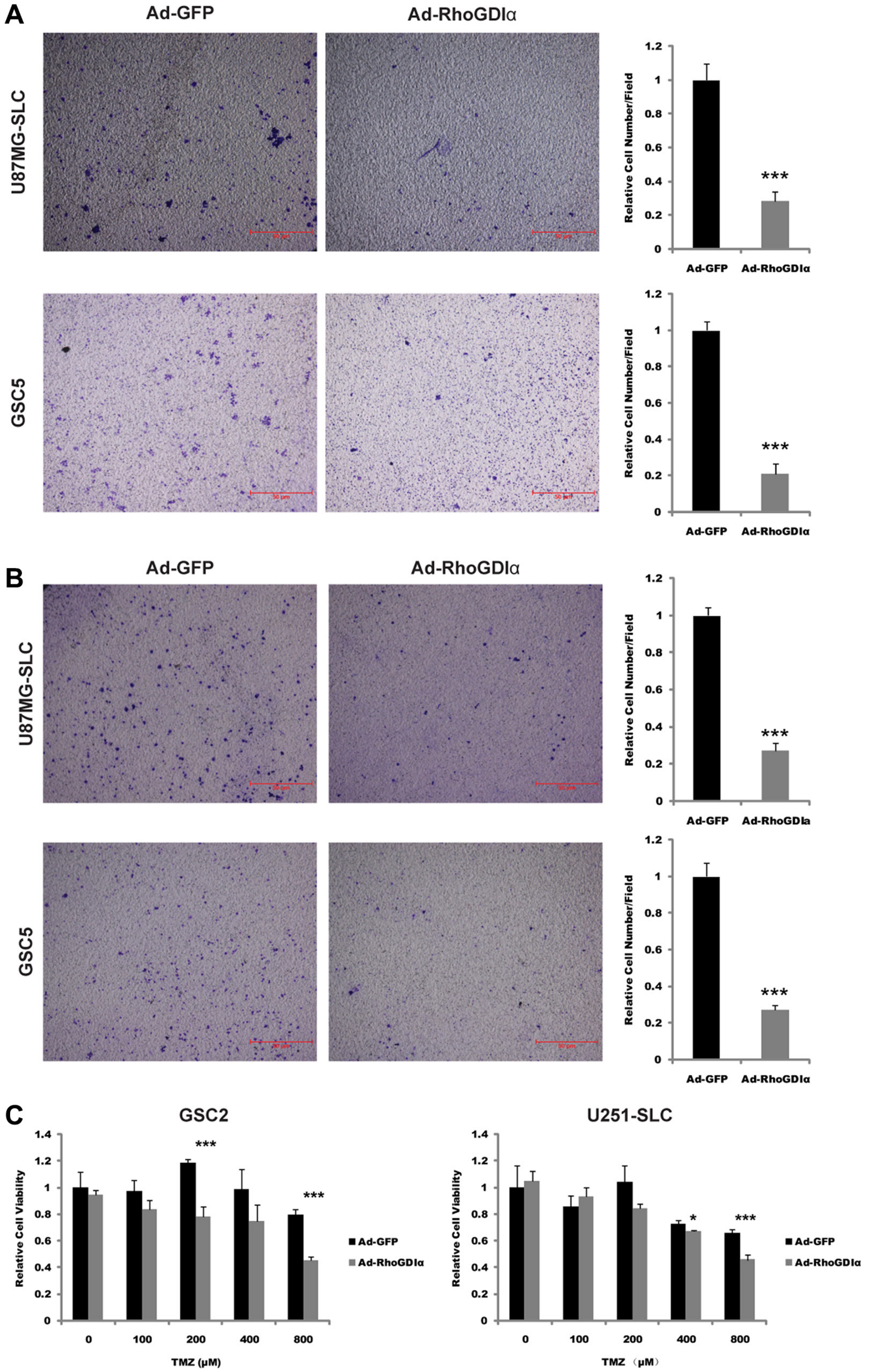

Supplementary Figure S5: RhoGDIα inhibited the cells migration and invasion and increased the TMZ sensitivity in GSCs. (**A**, **B**) The infected U87MG-SLC and GSC5 cells were suspended in 100 μl of neurobasal medium and loaded in the upper well of the 24-well transwell chamber. For invasion assay, the upper well was pre-coated with Matrigel (BD). After 48 hours of incubation, the Migrated and invaded cells were stained and counted. (**C**) GSC2 and U251-SLC cells were infected with Ad-RhoGDIα and Ad-GFP adenovirus (control), and then treated with TMZ at different concentrations. After 48 hours, the cells viability was detected by MTS assay. Data are means ± SD, *$p < 0.05$, **$p < 0.01$, ***$p < 0.001$.

Supplementary Table S1: Primer and siRNA sequences

RT-PCR primer	sequences (5′–3′)
SOX2	Forward: AAGAAAGGGAGAGAAGTTTGAGCC
	Reverse: GGCTCCGCGAGGAAAATC
Oct4	Forward: ACTGCAGCAGATCAGCCACATCGC
	Reverse: CTGCTTGATCGCTTGCCCTTCTGG
Nestin	Forward: GGCAGCGTTGGAACAGAGGT
	Reverse: CATCTTGAGGTGCGCCAGCT
CD133	Forward: GCATTGGCATCTTCTATGGTT
	Reverse: CGCCTTGTCCTTGGTAGTGT
Bmi1	Forward: TGGCCGCTTGGCTCGCATTCAT
	Reverse: ACAAAGCACACACATCAGGTGGGGA
RhoGDIα	Forward: TTTCCGCAGACCCCAACG
	Reverse: ATCTCTCGGTTAACCCGGAAAG
GAPDH	Forward: GGTCATCCATGACAACTTTGG
	Reverse: GGCCATCACGCCACAG
**Human Oct4 promoter primer**	Forward: CGCCTCGAGAGGATGGCAAGCTGAGAAACAC
	Reverse: TATAAGCTTGGGGAAGGAAGGCGCCCCAAG
**RhoGDIα siRNA**	siRNA-1: GGAAAGGCGUCAAGAUUGAdTdT
	siRNA-2: GAAGCAGUCGUUUGUGCUGdTdT

Supplementary Table S2: Clinical characteristics of GSC lines

GSC lines	Age (year)	Gender	Pathological diagnosis	Grade	Pathology information
GSC2	73	male	GBM with microvascular hyperplasy and necrosis	WHO IV	IDH1 (−), MGMT (−), PTEN (+), P53 (+), EGFR (+)
GSC5	31	male	Anaplastic oligoastrocytoma (right frontal lobe)	WHO III	IDH1 (−), MGMT (−), PTEN (+), P53 (+), EGFR (−)
